# Strengthening Personal Capacity of Older Adults in Culturally Diverse Context

**DOI:** 10.1093/geroni/igaf122.860

**Published:** 2025-12-31

**Authors:** Daniel W L Lai

## Abstract

The critical interplay of cultural diversity, digital access, lifelong learning, and personal capacity is a key focus, particularly for older adults in multicultural settings. These complex factors across diverse landscapes demand exploration to address challenges and enhance well-being. This symposium presents five studies examining personal capacity, social resilience, mental health, digital inclusion, and community empowerment among aging populations, spotlighting South Asian elders in Hong Kong, racialized immigrants in Canada, older adults in China, vulnerable seniors in Hong Kong’s digital context, and older Chinese immigrants in Canada. These studies underscore the need for culturally congruent, tech-savvy, and education-driven interventions. They highlight challenges in social connectivity, community engagement, mental health, digital literacy, and discrimination, alongside the power of intergenerational ties and learning. Critical elements like cross-cultural understanding, resilience depth, access to community, digital, and educational resources, family dynamics, and unmet care needs significantly shape older adults’ lives. The research strongly supports culturally attuned, digitally inclusive, and learning-focused policies and practices. It emphasizes empowerment, resilience-building, mental health support, digital capacity enhancement, and community advocacy tailored to diverse identities and needs. These findings are vital for crafting targeted interventions, policies, and frameworks to overcome unique barriers faced by ageing individuals in culturally diverse, digitally advancing, and socially evolving contexts worldwide. International Aging and Migration Interest Group Sponsored Symposium

